# Overexpression of *OsNAC14* Improves Drought Tolerance in Rice

**DOI:** 10.3389/fpls.2018.00310

**Published:** 2018-03-09

**Authors:** Jae Sung Shim, Nuri Oh, Pil Joong Chung, Youn Shic Kim, Yang Do Choi, Ju-Kon Kim

**Affiliations:** ^1^Graduate School of International Agricultural Technology and Crop Biotechnology Institute, GreenBio Science & Technology, Seoul National University, Pyeongchang, South Korea; ^2^Department of Agricultural Biotechnology, Seoul National University, Seoul, South Korea

**Keywords:** NAC transcription factors, OsNAC14, transgenic rice, drought tolerance, RNA-sequencing, ChIP

## Abstract

Plants have evolved to have sophisticated adaptation mechanisms to cope with drought stress by reprograming transcriptional networks through drought responsive transcription factors. NAM, ATAF1-2, and CUC2 (NAC) transcription factors are known to be associated with various developmental processes and stress tolerance. In this study, we functionally characterized the rice drought responsive transcription factor *OsNAC14*. *OsNAC14* was predominantly expressed at meiosis stage but is induced by drought, high salinity, ABA, and low temperature in leaves. Overexpression of *OsNAC14* resulted in drought tolerance at the vegetative stage of growth. Field drought tests demonstrated that *OsNAC14* overexpressing transgenic rice lines exhibited higher number of panicle and filling rate compared to non-transgenic plants under drought conditions. RNA-sequencing analysis revealed that *OsNAC14* overexpression elevated the expression of genes for stress response, DNA damage repair, defense related, and strigolactone biosynthesis. In addition, chromatin immunoprecipitation analysis confirmed the direct interaction of OsNAC14 with the promoter of *OsRAD51A1*, a key component in homologous recombination in DNA repair system. Collectively, these results indicate that OsNAC14 mediates drought tolerance by recruiting factors involved in DNA damage repair and defense response resulting in improved tolerance to drought.

## Introduction

Drought is a major environmental stress adversely affecting crop yield worldwide. Recent climate change increases occurrence and severity of drought stress in field. Moreover, the need to utilize farmland with inadequate water supply has substantially increased, as a consequence of global warming and world population growth. Thus, improving crop performance under drought conditions is an important objective in agriculture to support world food requirements. To cope with drought stress, plants have been evolved to possess molecular mechanisms that coordinate expression of genes to protect them from water deficit stress and increase the chance of survival in arid regions (Yamaguchi-Shinozaki and Shinozaki, 2006; Zheng et al., 2009).

Transcriptional reprograming required for drought tolerance mechanism is largely regulated by drought induced-transcription factors (TFs) such as AP2/ERF, MYB, bZIP, and NAC families (Yamaguchi-Shinozaki and Shinozaki, 2006; Nakashima et al., 2007; Zheng et al., 2009; Jung et al., 2017). Several studies have shown that overexpression of drought induced transcription factors can enhance drought tolerance. For example, overexpression of drought induced *OsERF48, OsERF71, OsMYB2, OsbZIP12*, and *OsbZIP71* enhance drought tolerance in rice (Yang et al., 2012; Joo et al., 2014; Liu et al., 2014; Lee et al., 2016; Jung et al., 2017). Thus, identification and characterization of drought induced TFs is useful approach to understand molecular mechanisms underlying in drought tolerance and generate plants showing enhanced performance under drought condition (Jung et al., 2017; Lee D. K. et al., 2017).

NAC transcription factor family is one of the largest families of plant-specific transcription factors, named from first three reported members of the family, petunia NO APICAL MERISTEM (NAM), Arabidopsis ACTIVATION FACTOR (ATAF), and CUP-SHAPED COTYLEDON (CUC) (Souer et al., 1996; Aida et al., 1997). Genome-wide analysis has identified 117 NACs in Arabidopsis, 151 in rice, 79 in grape, 163 in poplar, 152 each in soybean, and tobacco (Ooka et al., 2003; Nuruzzaman et al., 2010, 2013). NAC proteins contain a highly conserved N-terminal DNA-binding domain that can form either a homodimer or a heterodimer and a highly variable C-terminal region (Ooka et al., 2003; Zheng et al., 2009). NACs were originally identified as key regulators of development through forward genetic screens (Souer et al., 1996; Takada et al., 2001). Recently, NACs have been reported to regulate wide range of abiotic stress responses in plants (Nuruzzaman et al., 2013). In Arabidopsis, *AtNAC72* (*RD26*), *AtNAC19*, and *AtNAC55* are required for drought tolerance (Fujita et al., 2004; Tran et al., 2004). Overexpression of *ATAF1* enhances drought tolerance through stomatal closure in Arabidopsis (Wu et al., 2009). In rice, the members in stress-induced NAC (SNAC) subgroup are reported as key regulators of drought tolerance in rice. Overexpression of *OsNAC5, OsNAC6, OsNAC9*, and *OsNAC10* confers drought tolerance via root structural adaptation and up-regulation of genes involved in stress responses, redox homeostasis, defense responses, and ABA biosynthesis (Nakashima et al., 2007; Jeong et al., 2010, 2013; Takasaki et al., 2010; Redillas et al., 2012; Lee D. K. et al., 2017).

Maintenance of genome stability is another important process in plants under abiotic stress for survival and faithful transmission of genetic information to next generation (Tuteja et al., 2001; Roy, 2014). Plants are subjected to high levels of DNA damage resulting from exposure to environmental stresses such as cold, high temperature, UV-C, and drought (Tuteja et al., 2001; Roldan-Arjona and Ariza, 2009; Roy, 2014). Reactive oxygen species (ROS) generation is one potential cause of DNA damage under drought conditions because drought induced DNA damage is alleviated by exogenous application of ROS scavenger (Tuteja et al., 2001; Wang and Zhang, 2001). Among various forms of DNA lesions generated by drought stress, double strand breaks (DSBs) in DNA are considered as one of the major form of DNA damage (Yao et al., 2013; Roy, 2014). DSBs are repaired by homologous recombination mediated by Radiation sensitive 51 (RAD51) and Mre11-RAD50-Nbs1 (MRN) complex (Shinohara et al., 1992; Symington, 2002). Function of RAD51 in homologous recombination is highly conserved in various organisms (Shinohara et al., 1992; Li et al., 2004, 2007; Khoo et al., 2008). Failure of DNA repair leads to deterioration of cell function and cell death (Tuteja et al., 2001). Thus, proper regulation of DNA repair is required for drought tolerance. However, the underlying molecular mechanisms are still elusive.

In this study, we investigated the molecular mechanism of *OsNAC14*-mediated drought tolerance responses. OsNAC14 belongs to ONACII subgroup of Group A NAC TFs. Transgenic rice overexpressing *OsNAC14* exhibited enhanced drought tolerance at the vegetative and the reproductive stages of growth. We also identified downstream target genes constituting the OsNAC14-mediated drought tolerance pathway which are involved in stress response, DNA repair, defense-related and strigolactone biosynthesis. OsNAC14 was found to regulate DNA repair pathway by directly regulating the homologous recombination component *OsRAD51A1*. These data suggest that OsNAC14 regulates *OsRAD51A1* which in turn enhances drought tolerance of plants.

## Materials and methods

### Plasmid construction and rice transformation

The coding region of *OsNAC14* (*Os01g0675800*) was amplified from rice (*Oryza sativa* cv. Nipponbare) total RNA using the Reverse Transcription System (Promega) and PrimeSTAR HS DNA polymerase (TAKARA). The amplified OsNAC14 coding sequence was cloned into rice transformation vector p700 carrying *PGD1* promoter for constitutive overexpression (Park et al., 2012). The final construct named *PGD1::OsNAC14* was transformed into rice (*Oryza sativa* cv. Nakdong) by Agrobacterium (LBA4404)-mediated co-cultivation, as described previously (Jang et al., 1999). Copy numbers *PGD1::OsNAC14* transgenic plants were determined by TaqMan Q-PCR (ThermoFisher) using probes specific for the bar gene. To analyze copy number of the transgenic rice plants, genomic DNA was extracted from 2-week-old rice seedlings. Genomic DNA extracted from transgenic plants previously confirmed as single inserted homozygous line was used as control. The single copy insertion lines self-fertilized and homozygous transgenic lines were selected from T_2_ generations on MS media containing phosphinothricin (Duchefa).

For CRISPR/Cas9-mediated *OsNAC14* mutagenesis, the CRISPR/Cas9 expression vector was constructed using a rice codon-optimized *Streptococcus pyogenes* Cas9 (rCRISPR/Cas9) and guide RNA (gRNA) targeting CDS region (700–722 bp) of OsNAC14 in pSB11 vector through restriction enzyme-mediated excision and ligation reactions. To generate rice codon-optimized Cas9, the original Cas9 sequence was changed to a sequence suitable for translation in rice. Then, rice codon-optimized Cas9 was chemically synthesized (Bioneer, Korea), and used for further constructions. Nuclear localization sequence (NLS) was fused to both N-terminus and C-terminus of rCRISPR/Cas9, and self-cleaving 2A peptide (P2A), and GFP were inserted between rCRISPR/Cas9 and C-terminal NLS sequence. For the gRNA cassettes in these vectors, the rice *U6* promoter and a custom designed gRNA (5′- AAGAGCTCTGGTGCAAGAAGGGG-3′) targeting CDS region (700–720 bp) of *OsNAC14* were introduced into pSB11 vector through following procedures. To minimize the possibility of off-target effects potentially caused by CRISPR/Cas9 mediated mutagenesis, we performed computational analysis to select unique and specific sequence which can be used for gRNA target site on *OsNAC14* coding sequence using CRISPRdirect software (https://crispr.dbcls.jp/). Rice *U6* promoter and 5′-region of gRNA was amplified by PCR reactions (F: 5′-CCCAAGCTTAAGGAATCTTTAAACATACGA-3′, R: 5′-CTTCTTGCACCAGAGCTCTTGCCACGGATCATCTGCA). 3′-region of gRNA was amplified with 20 base overlap with 5′region of gRNA by PCR reactions (F:5′-AAGAGCTCTGGTGCAAGAAGGTTTTAGAGCTAGAAATAGG-3′, R:5′- TGCTCTAGAAAAACAAAAAAGCACCGACTCGGTGC-3′). Two PCR products were mixed and used as template for crossover PCR using primers recognizing 5′-region of *U6* promoter and 3′-region of gRNA (F: 5′-CCCAAGCTTAAGGAATCTTTAAACATACGA-3′, R:5′- TGCTCTAGAAAAACAAAAAAGCACCGACTCGGTGC-3′). Final PCR products were digested with HindIII and XbaI, and inserted into pSB11 vector by ligation. The plasmid was introduced into rice (*Oryza sativa* cv. Dongjin) using Agrobacterium-mediated co-cultivation method. Primer sequences used in this study are listed in Table S2.

### Drought stress treatment and tolerance evaluation

Transgenic and NT (non-transgenic) (*Oryza sativa* cv. Nakdong) seeds were germinated on Murashige and Skoog (MS) medium (Duchefa Biochemie) with 3% sucrose in the dark for 3 days at 28°C, and transferred into light conditions for 1 day. Thirty Seedlings from each transgenic and NT plants were transplanted in soil pot (4 × 4 × 6 cm, three plants per pot) and grown for 5 weeks in the greenhouse (16 h-light/8 h-dark cycle) at 30°C. Drought stress was imposed by sequentially withholding water for 3 days and re-watering for 5 days. Drought-induced symptoms were visualized by imaging tested plants at indicated time point using an NEX-5N camera (Sony), and soil moisture was measured at indicated time point using a SM 150 soil moisture sensor (Delta T Devices).

Transient chlorophyll *a* fluorescence and the performance index were measured using a HANDY PEA fluorimeter (Hansatech Instruments), as described previously (Jung et al., 2017). Two-week-old plants were transplanted in soil pot (15 × 15 × 14 cm) and grown for 5 weeks. Chlorophyll fluorescence and the performance index were measured from longest leaves of each plant after 1 h of dark adaptation to ensure sufficient opening of the reaction center. Measurement was performed at apex, middle, and base regions of leaves using the Handy-pea fluorimeter (Hansatech Instrument). Thirty readings per line were averaged using the HANDY-PEA software (version 1.31). Fv/Fm value and the performance index were calculated according to the equations of the JIP test (Redillas et al., 2011).

### Evaluation of the agronomic traits of rice plants grown in the field

To Evaluate yield components of transgenic and non-transgenic (NT) plants under normal field conditions, three independent T4 homozygous lines of the *OsNAC14*^*OX*^ plants and NT plants were planted in the rice paddy field at Kyungpook National University, Gunwi (36°06′48.0″N,128°38038.0″E), Korea (RDA-A-2011-005, 2016). Yield parameters were scored from 30 plants collected from three different plots for normal field conditions. To evaluate yield components of the plants under drought field conditions, plants were grown in semi-field conditions under rain-off shelters before drought treatment. Intermittent drought stress was applied twice by withholding water during panicle development stage. Drought treatment was monitored by measuring soil water content using Soil Moisture Sensor (AT Delta-T Device). After two rounds of drought treatment, the plants were irrigated until harvesting. Yield components were scored from 18 plants for each line for drought field conditions. The results were compared between transgenic and NT plants using ANOVA (*p* < 0.05 level) with Fisher's least significant difference for multiple comparisons.

### RNA-sequencing analysis

Total RNA was extracted from rice leaves (2 weeks old, grown soil) using Trizol reagent (Invitrogen) and purified on-colum DNase treatment with RNeasy Mini Kit (Qiagen). The libraries were prepared using the TruSeq RNA sample Prep Kit (v2) (Macrogen). RNA-sequencing was repeated twice with samples from NT and *PGD1::OsNAC14* transgenic plants. Single-end sequences were obtained using IRGSP (v 1.0) and raw sequence reads were trimmed to remove adaptor sequences, and those with a quality lower than Q20 were removed using the Trimmomatic 0.32 software (Bolger et al., 2014). To map the reads to reference genome, all reads were assembled with annotated genes from the Rap-DB database [http://rapdb.dna.affrc.go.jp; IRGSP (v 1.0)] using TopHat software (https://ccb.jhu.edu/software/tophat/index.shtml). After mapping reads to a reference genome differentially expressed genes were analyzed and validated by more than two-fold change value and independent *T*-test (*p*-value < 0.05), then 554 transcripts were selected for further analysis. The data set can be found at from GEO database with series accession number GSE106150 for RNA-sequencing data (http://www.ncbi.nlm.nih.gov/geo/).

### Real-time PCR analysis

Total RNA was extracted from *OsNAC14*^*OX*^ transgenic (Line 11) and NT plants grown for 2 weeks in soil using Trizol reagent (Invitrogen) according to the manufacturer's instructions. To generate first-strand complementary DNA (cDNA), 1 μg of total RNA was reverse-transcribed using RevertAid M-MuLV Reverse Transcriptase (Thermo Scientific). Subsequent quantitative real time PCR (qRT-PCR) was performed with 2X Real-Time PCR smart mix (SRH72-M10h, SolGent) and EvaGreen (31000-B500, SolGent). The PCR reactions were performed by initial denaturation at 95°C for 15 min, followed by 40 cycles of 95°C for 20 s, 60°C for 20 s, and 72°C for 30 s, using Strategene Mx300p real-time PCR machine (Stratagene). *UBIQUITIN1* (Os06g0681400) was used as internal control for normalization. Three biological replicates were analyzed for quantitative experiments. The primer information used for qRT-PCR gene expression is listed in Table S2.

### Protoplast isolation and transient gene expression

Polyethylene Glycol (PEG)-mediated protoplast transformation system was used to transiently express *OsNAC14* and verify the correlation between *OsNAC14* and its target genes (Chen et al., 2006; Yoo et al., 2007; Zhang Y. et al., 2011). Rice seedlings (*Oryza sativa* cv. Ilmi) were grown in the dark for 10 d and transferred to the light conditions for 8~10 h. Leaf sheaths of 100 rice seedlings were cut into 0.5 mm pieces using a sharp blade on a glass. The pieces were transferred into 0.6 M mannitol solution and incubated for 30 min at room temperature in the dark conditions. After removal of mannitol solution, the pieces were soaked in enzyme solution [1.5% Cellulase R-10 (Yakult, Japan), 0.75% Macerozyme R-10 (Yakult, Japan), 0.5 M mannitol, 10 mM MES (pH 5.7), 0.1% BSA, 10 mM CaCl_2_, and 5 mM β-mercaptoethanol] for cell wall degradation. Vacuum infiltration was applied to the enzyme solution for 15 min using desiccator and the digestion was carried out in the dark at 28°C for 4 h with gentle shaking. The enzyme solution was filtered twice through 70 μm and 40 μm nylon meshes (Falcon, USA). The flow-through was centrifuged at 300 g and the protoplast pellet was resuspended in W5 solution [154 mM NaCl, 125 mM CaCl_2_, 5 mM KCl, 2 mM MES (pH 5.7)]. The protoplast concentration was measured under the microscope using a hemocytometer (Marienfeld) and adjusted to 7.0 × 10^7^ protoplasts/mL. Fifty microliters of protoplasts (2.5 × 10^6^ cells) was mixed with 15 μL of plasmids and 130 μL of PEG solution. The mixture was incubated for 15 min at 28°C in the dark. After incubation, 1 mL of W5 solution was added into mixture, and centrifuged at 300 g for 2 min to collect protoplasts. The protoplasts were resuspended in Incubation solution for 10 hr. Protoplasts were harvested by centrifugation at 300 g for 2 min and used for RNA extraction. Relative expression levels of genes in protoplasts were analyzed using qRT-PCR analysis. Detailed conditions for RNA extraction and qRT-PCR analysis are described in “Real-time PCR analysis” section.

### Subcellular localization of *OsNAC14*

The coding region of *OsNAC14* amplified from the cDNA was cloned into the pHBT vector carrying GFP-myc using the In-fusion system (Clonetech). The final construct (*35S::OsNAC14-GFP*) and the control vectors (*35S::GFP*) were transfected into protoplasts (*Oryza sativa* cv. Ilmi) using PEG-mediated protoplast transformation system. *35S::NF-YA7-GFP* was used as control for nuclear localization (Lee et al., 2015). GFP and mCherry signals were observed 12 h after transfection using SP8 STED confocal fluorescence microscope (Leica).

### Chromatin immunoprecipitation (ChIP) assay

ChIP assay was performed according to Bowler et al. (2004) with minor modifications (Bowler et al., 2004). The transfected protoplasts (5–7 × 10^7^ protoplasts/one tube) were cross-linked with 1% formaldehyde by vacuum infiltration for 15 min, and the cross-linking reaction was stopped by the addition of 2 M glycine to a final concentration of 125 mM. The protoplasts pellet was resuspended in 10 ml of pre-chilled extraction buffer 1 [0.4 M Sucrose, 10 mM Tris-HCl (pH 8.0), 5 mM β-mercaptoethanol, protease inhibitors] for 30 min in the ice with gentle agitation. After centrifugation for 20 min at 2,880 g at 4°C, the pellet was resuspended in extraction buffer 2 [0.25 M Sucrose, 10 mM Tris-HCl (pH 8.0), 10 mM MgCl_2_, 1% Triton X-100, 5 mM β-mercaptoethanol, protease inhibitors]. After subsequent centrifugation for 10 min at 12,000 g at 4°C, the pellet was resuspended in extraction buffer 3 [1.7 M Sucrose, 10 mM Tris-HCl (pH 8.0), 0.15% Triton X-100, 2 mM MgCl_2_, 5 mM β-mercaptoethanol, 0.1 mM PMSF, protease inhibitors] and loaded on the extraction buffer 3 (300 ul). After centrifugation for 60 min at 16,000 g at 4°C, the chromatin pellet was resuspended in nuclei lysis buffer [50 mM Tris-HCl (pH 8.0), 10 mM EDTA, protease inhibitors]. To shear DNA to ~100–200 bps DNA fragments, the chromatin solution was sonicated in ice using Bioruptor (Diagenode) for 20 min with 30 s ON and 30 s OFF cycle. The sonicated chromatin extract was centrifuged for 5 min at 12,000 g at 4°C, and the supernatant was diluted 10 times with ChIP dilution buffer [1.1% Triton X-100, 1.2 mM EDTA, 16.7 mM Tris-HCl (pH 8.0), 167 mM NaCl, protease inhibitors]. Diluted chromatin extract was incubated with anti-myc polyclonal antibody (sc789x, Santa Cruz) or without antibody overnight at 4°C, then, further incubated with protein A agarose beads with gentle agitation for 2 h. The beads were washed three times with low salt, high salt, LiCl, and TE wash buffers. The pellet was resuspended in elution buffer [1% SDS, 0.1 M NaHCO_3_] and incubated at 65°C for 15 min. The resultant eluted DNAs were purified using the QIAquick PCR purification kit (Qiagen). The purified DNA was used in quantitative real-time PCR reactions using Mx3000P Real-Time PCR system (Agilent Technologies). To determine the positions of NAC binding motif on *OsRAD51A1* and *Piz-t* promoter region, the promoters were analyzed using A Database of Plant Cis-acting Regulatory DNA Elements (https://sogo.dna.affrc.go.jp/cgi-bin/sogo.cgi?lang=en&pj=640&action=page&page=newplace). Five sets of primers were designed based on the positions of NAC binding motif on *OsRAD51A1* and *Piz-t* promoter for ChIP-PCR analysis. The relative enrichment was normalized against the 1% of total input DNA.

## Results

### OsNAC14 is a drought-inducible transcription factor

Phylogenetic analysis revealed that OsNAC14 belongs to the ONACII family of group A. Members of this group are separated from the well-characterized stress-associated NAC (SNAC) family in terms of its diversity in NAC domain structure (Figure S1A) (Nuruzzaman et al., 2010). In group A, 29 members are responsive to water deficit conditions in leaves (Chung et al., 2016) suggesting that ONACII genes, including *OsNAC14*, may be another component of the molecular pathway to regulate drought responses of plants.

**Figure 1:**
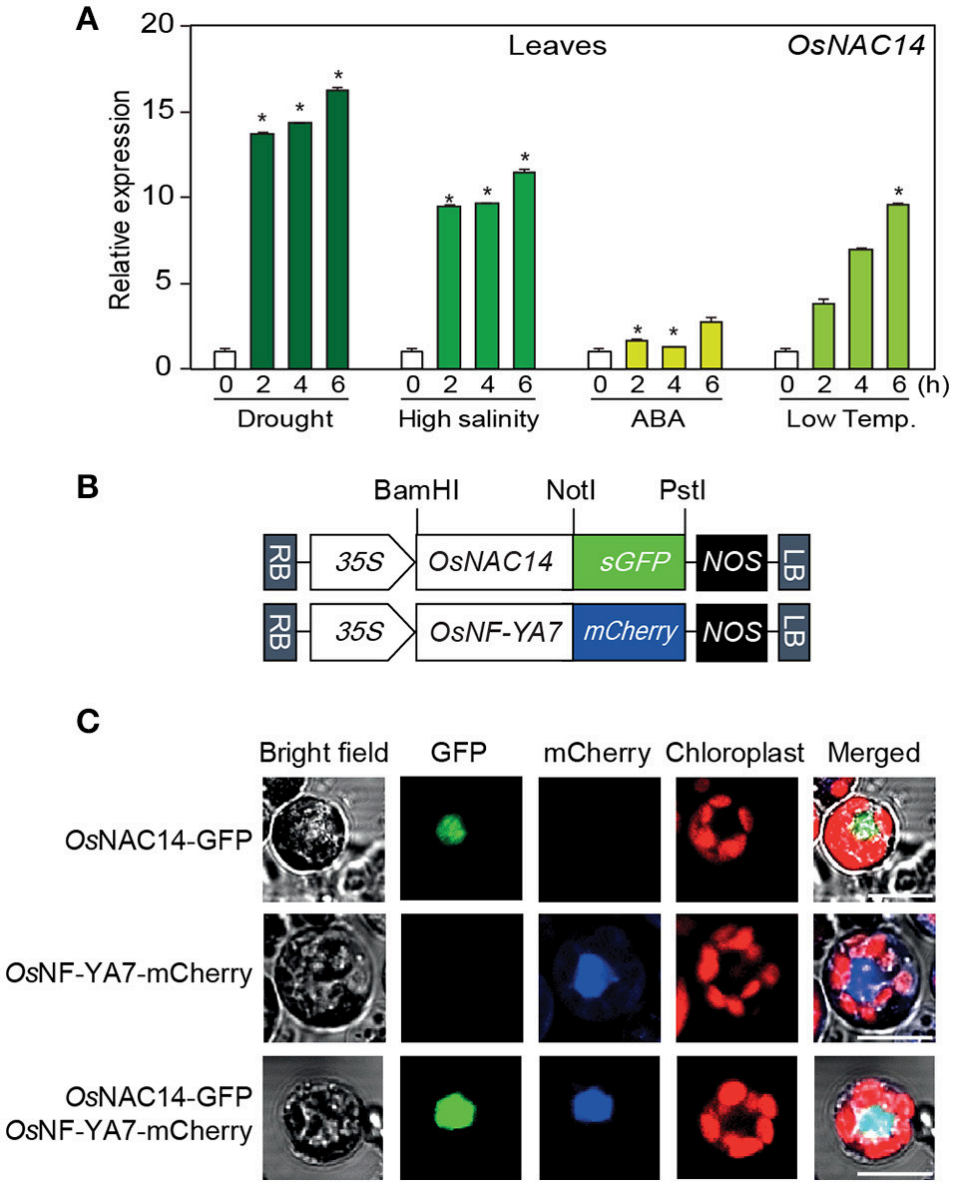
Characterization of *OsNAC14*. **(A)** The relative expression patterns of *OsNAC14* in response to four different abiotic stresses. Two-week-old rice seedlings (*Oryza sativa*. L. Japonica cv. Ilmi) were exposed to air-drying (drought), 400 mM NaCl (high salinity), 100 μM abscisic acid (ABA), and 4°C (low-temperature). Leaves of rice plants were harvested at indicated time point after treatment. *OsUBIQUITIN1* (*OsUbi1*) was used as internal control for normalization. Data represent mean value + standard deviation (*SD*) (*n* = 3). Significant differences from non-treated control are indicated by asterisks (one-tailed Student's *t*-test, ^*^P < 0.05). **(B,C)** Subcellular localization of OsNAC14 in rice protoplast. **(B)** Schematic diagram of OsNAC14-GFP and OsNF-YA7-mCherry expression constructs **(C)** protoplasts were transiently co-transfected with OsNAC14-GFP and OsNF-YA7-mCherry expression constructs. Fluorescence was observed in protoplasts 12 h after transfection using a confocal microscope. Scale Bar = 10 μm.

To investigate the functions of *OsNAC14* in drought stress response, we shortlisted a number of genes from our previously reported microarray data on leaves of rice exposed to different stresses such as drought, high salinity, abscisic acid (ABA), and low temperature (Oh et al., 2009). From these, we found that *OsNAC14* was highly induced by drought and high-salinity treatments (Figure S1B). The drought-inducible expression pattern of *OsNAC14* was further confirmed from an independent RNA-sequencing experiment performed by Chung et al. (2016) (Figure S1C). Similarly, *OsNAC14* was up-regulated over a time course in response to drought stress (Figure S1C). *OsNAC*s belonging to SNAC subfamily (*OsNAC5, OsNAC6, OsNAC9*, and *OsNAC10*), previously reported as drought-inducible *OsNAC* transcription factors, were also up-regulated in response to drought stress (Figure S1C). Transcripts of *OsNAC9* and *OsNAC14* were increased later than that of *OsNAC5* and *OsNAC6* in response to drought stress (Figure S1C). The responses of *OsNAC14* transcription in stress conditions were further verified through quantitative real-time polymerase chain reaction (qRT-PCR) using total RNA from leaves and roots collected from 2-week-old rice seedlings (*Oryza sativa* L. cv. Ilmi) exposed to drought, high salt, abscisic acid (ABA), and low temperature (Figure 1A and Figure S2). *OsNAC14* expression was strongly induced within 2 h of exposure to drought, high salt, ABA, and low temperature stresses in leaves. The induction of *OsNAC14* expression in stress conditions were predominant in leaves (Figure 1A) than in root tissues (Figure S2).

To further characterize the function of OsNAC14, we performed a subcellular localization analysis to confirm its nuclear localization due to the nuclear localization sequence found at the C-terminal side of its sequence (Kosugi et al., 2009) (Figure S3). We generated a construct to express translationally fused OsNAC14 and GFP fluorescent protein (GFP) (OsNAC14-GFP) driven by the CaMV 35S promoter and transiently expressed in rice protoplasts together with OsNF-YA7-mcherry as positive control for nuclear localization (Lee et al., 2015) (Figure 1B). The fluorescence signals from both GFP and mCherry were co-localized in the nuclear region confirming that OsNAC14 is a nuclear-localized protein (Figure 1C). Taken together, these results suggest that OsNAC14 is responsive to drought stress and is localized in the nucleus of the cell.

### Overexpression of *OsNAC14* improves drought tolerance at the vegetative stage

**Figure 2:**
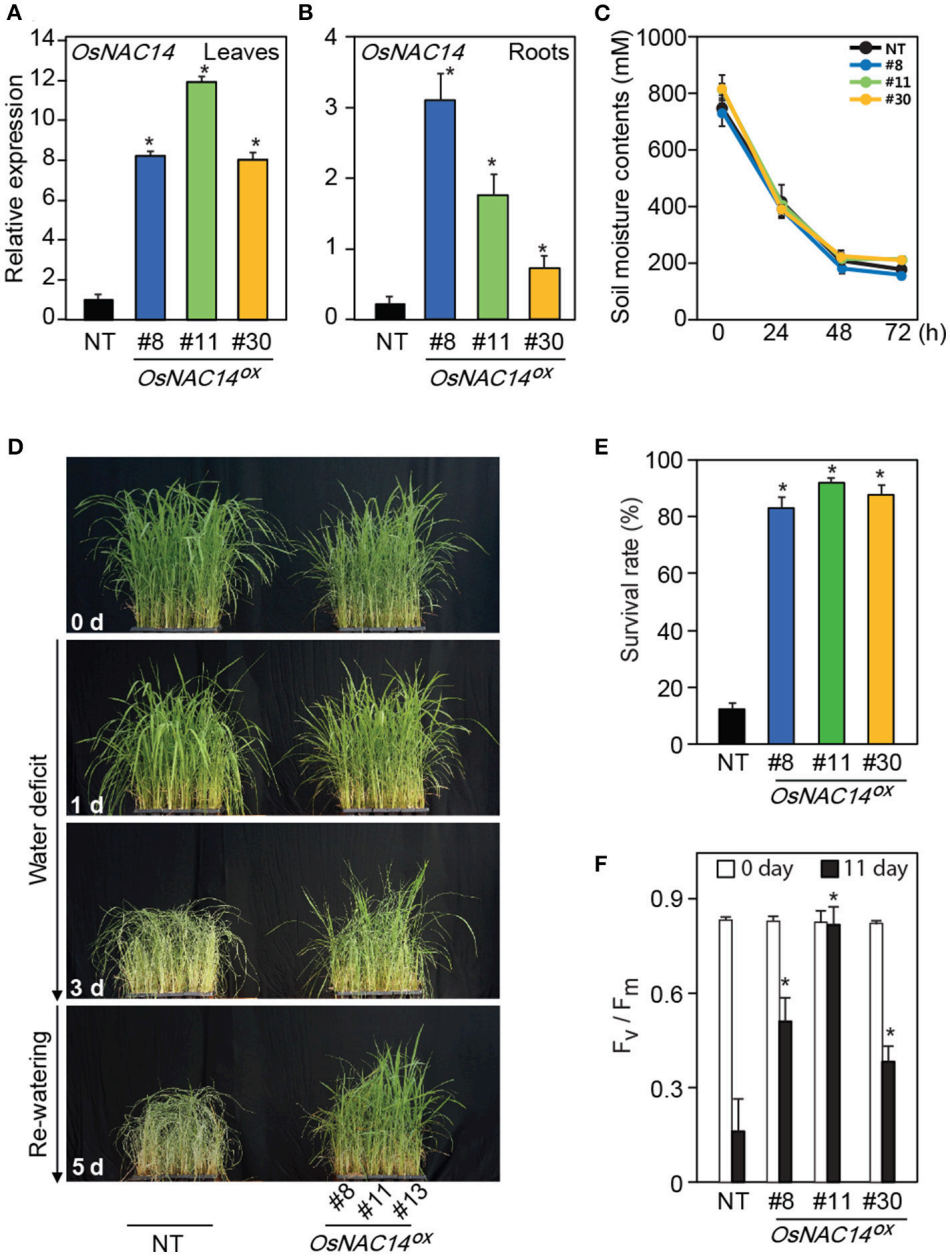
*OsNAC14* overexpression in rice enhances drought resistance**. (A,B)** Relative expression levels of *OsNAC14* in non-transgenic (NT) and three independent T4 homozygous lines of *PGD1::OsNAC14* (*OsNAC14*^*OX*^) plants. Total RNAs were extracted from leaves **(A)** and roots **(B)** of two-week-old rice seedlings. *OsUbi1* was used as internal control for normalization. Data represent mean value + *SD* (*n* = 3). **(C)** Measurement of soil moisture contents (mV). Data represent mean value ± *SD* of 30 measurements performed at different locations of soil. **(D)** The phenotype of transgenic rice plants during drought stress. Five-week-old three independent T4 homozygous lines of *OsNAC14*^*OX*^ plants and NT were exposed to drought stress for 3 days, followed by re-watering. Numbers on the image indicate duration of drought treatment and re-watering. **(E)** The survival rate of transgenic plants 5 days after re-watering. Data represent mean value + SD (*n* = 30). **(F)** Chlorophyll fluorescence (Fv/Fm) contents of plants under drought condition. Five-week-old three independent T4 homozygous lines of *OsNAC14*^*OX*^ and NT plants were exposed to drought stress for 11 days. Chlorophyll fluorescence was measured in the dark at indicated time point using a Pulse Amplitude Modulation (PAM) fluorometer. Data represent mean value + *SD* (*n* = 20). Significant differences from NT control are indicated by asterisks (one-tailed Student's *t*-test, ^*^P < 0.05).

To investigate the physiological functions of *OsNAC14* in plants in response to drought, transgenic rice plants overexpressing *OsNAC14* were generated by transforming *PGD1*::*OsNAC14* into *Nakdong* cultivar (designated as *OsNAC14*^*OX*^). Thirty independent lines were produced and to avoid the effects of somaclonal variations, plants that grew normally without stunting were screened out. Based on the expression levels of *OsNAC14* (Figures 2A,B) we finally selected three independent homozygous lines (#8, #11, and #30).

To compare the performance of plants under drought conditions, the selected *OsNAC14*^*OX*^ and non-transgenic plants (NT, Nakdong) were grown in a greenhouse for 5 weeks and exposed to drought conditions by withholding water for 3 days and monitored drought-induced visual symptoms (Figures 2C–E). Soil moisture contents showed a consistent rate of decrease among different pots indicating that stress treatments were uniformly applied to the plants (Figure 2C). Drought-associated symptoms, such as leaf rolling, wilting, and loss of chlorophyll appeared earlier in NT plants than in *OsNAC14*^*OX*^ plants during drought treatment (Figure 2D). Transgenic plants also showed faster recovery compared to NT after being relieved from drought stress through re-watering (Figures 2D,E). *OsNAC14*^*OX*^ plants scored 5 days after re-watering showed 83 to 92% survival rate whereas NT plants only showed 12% (Figure 2E). To further verify the stress tolerant phenotype of the plants, Fv/Fm values, an indicator of the photochemical efficiency of photosystem II, were measured in plants grown in bigger pots and exposed to drought for 11 days (Figure 2F and Figure S4). Similar gradual decrease in soil moisture contents between pots were observed showing uniform stress treatment (Figures S4A,B). The Fv/Fm values in NT plants showed a more rapid decrease than in *OsNAC14*^*OX*^ plants during drought treatments (Figure 2F and Figure S4C). Taken together, these results suggest that overexpression of *OsNAC14* enhances drought tolerance of plants when exposed to drought.

### *OsNAC14* is significantly expressed during meiosis

**Figure 3:**
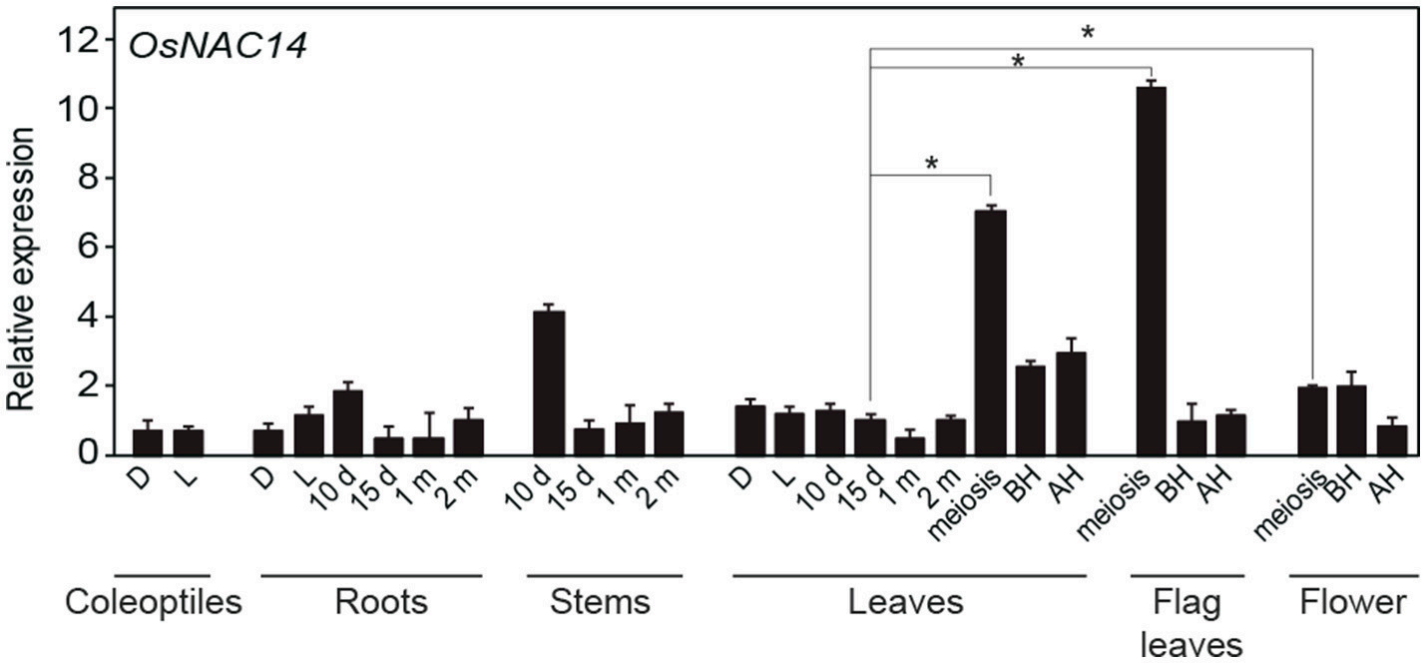
*OsNAC14* expression at various developmental stages. qRT-PCR analysis of *OsNAC14* expression in rice tissues at different developmental stages (*Oryza sativa*. L. Japonica cv. Ilmi). d, day; D, Dark; BH, Before heading; AH, After heading. *OsUbi1* was used as internal control for normalization. Data represent mean value + standard deviation (*SD*) (*n* = 3). Significant differences from 15 days old leaves tissue are indicated by asterisks (one-tailed Student's *t*-test, ^*^P < 0.05).

Since NAC transcription factors are known to regulate various developmental processes in plants (Souer et al., 1996; Aida et al., 1997) we examined the expression levels of *OsNAC14* transcripts in various rice tissues at different developmental stages starting from coleoptile to flower formation (Figure 3). qRT-PCR analysis showed that *OsNAC14* transcripts were detected from all the tested tissues. The highest expression of *OsNAC14* was detected during meiosis stage in both leaves and flag leaves. These suggest the OsNAC14 might be involved in processes occurring during the meiosis stage.

### Effect of *OsNAC14* overexpression on grain yield under field drought conditions

Since grain production is seriously affected by drought stresses at the reproductive stage of growth, we evaluated yield components of *OsNAC14*^*OX*^ plants in year 2016 in a rice paddy field. Three independent T_4_ homozygous *OsNAC14*^*OX*^ transgenic and NT plants were planted in a paddy field and grown to maturity. Under normal conditions, *OsNAC14*^*OX*^ plants exhibited reductions in panicle length (−0.65 to −5.36%) and number of total grains (−2.24 to −17.93%) (Table 1). In the drought conditions, however, the overexpression of *OsNAC14* resulted in higher filling rate (12.25 to 36.64%) and number of panicles (5.88 to 18.62%) though there was a decrease in panicle length (−2.48 to −14.89%) and number of total grain (−2.24 to −27.34%) compared to NT (Table 1).

**Table 1:** Agronomic traits of *OsNAC14*^*OX*^ transgenic plants grown in the field.

**Normal**		**Panicle length (cm)**	**No. of panicle (/hill)**	**No of total grain (/hill)**	**Filling rate (%)**	**1000 grain weight (g)**
NT	Average	20.47	17.10	1688.19	87.62	24.60
*OsNAC14*^*OX*^ #8	Average	19.37	16.54	1385.44	87.12	24.24
	%Δ	−5.36	−3.28	−17.93	−0.57	−1.50
	*p*-value	0.00^**^	0.65	0.15	0.79	0.41
*OsNAC14*^*OX*^ #11	Average	20.33	14.17	1650.44	86.96	25.41
	%Δ	−0.65	−17.14	−2.24	−0.75	3.28
	*p*-value	0.74	0.01^*^	0.93	0.73	0.04^*^
*OsNAC14*^*OX*^ #30	Average	20.00	18.13	1468.33	90.55	23.85
	%Δ	−2.28	6.02	−13.02	3.34	−3.08
	*p*-value	0.25	0.38	0.31	0.13	0.35
**Drought**		**Panicle length (cm)**	**No. of panicle (/hill)**	**No of total grain (/hill)**	**Filling rate (%)**	**1000 seed weight**
NT	Average	17.63	19.13	1751.44	33.62	18.72
*OsNAC14*^*OX*^ #8	Average	15.00	21.63	1272.56	37.74	19.17
	%Δ	−14.89	13.07	−27.34	12.25	2.41
	*p*-value	0.00^**^	0.14	0.00^**^	0.24	0.02^*^
*OsNAC14*^*OX*^ #11	Average	17.19	20.25	1601.69	45.87	19.23
	%Δ	−2.48	5.88	−2.24	36.44	2.73
	*p*-value	0.49	0.51	0.93	0.03^*^	0.05
*OsNAC14*^*OX*^ #30	Average	16.5	22.69	1468.33	43.81	18.52
	%Δ	−6.38	18.62	−13.02	30.31	−1.07
	*p*-value	0.08	0.04^*^	0.13	0.08	0.01

Panicle length, number of panicle, number of grain, filling rate, and 1000 seed weight of three independent lines of OsNAC14^OX^ transgenic rice plants. Percentage differences (%Δ) between the values for the OsNAC14^OX^ transgenic rice plants and NT controls are listed. An asterisk indicates a significant difference (

* P < 0.05 and

** *P < 0.01)*.

### Identification of genes regulated by *OsNAC14*

**Figure 4:**
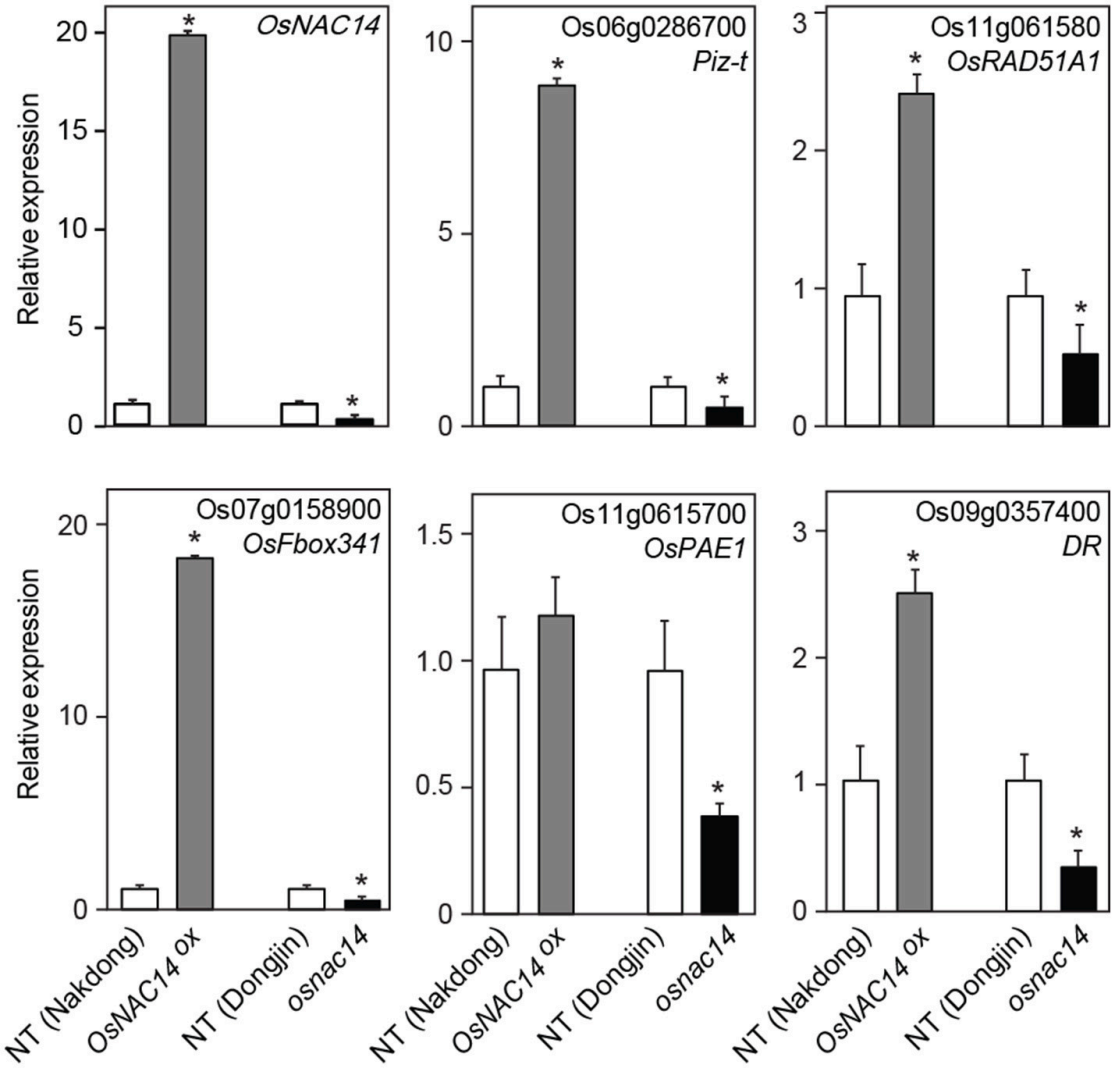
Expression of differentially expressed genes (DEGs) isolated from RNA-sequencing data. qRT-PCR analysis of DEGs in NT, *OsNAC14*^*OX*^, and *osnac14* mutant plants. *OsUbi1* was used as internal control for normalization. Data represent mean value + standard deviation (*SD*) (*n* = 3). Significant differences from NT control are indicated by asterisks (one-tailed Student's *t*-test, ^*^P < 0.05).

To understand the transcriptional network regulated by the *OsNAC14* during drought, RNA-sequencing analysis was performed on 2-week-old *OsNAC14*^*OX*^ plant (T4 generation, line 11) grown under normal growth conditions (Table 2). From the 37,972 differentially expressed genes (DEG) we then selected those that showed at least two-fold change (*p*-value < 0.05). The analysis revealed 122 up-regulated and 151 down-regulated genes following the overexpression of *OsNAC14* relative to NT (Table S1). These genes were then processed for Gene Ontology analysis using PANTHER Classification System (http://pantherdb.org) (Figure S5). Results revealed that majority of the genes were assigned to catalytic activity (62.7%) under the molecular function class and to metabolic process (32.0%) and cellular process (29.0%) pathway under the biological class. Among these, 23 up-regulated genes were further selected through their gene annotation and reported studies on stress response (Table 2). These up-regulated genes were associated with stress response, DNA damage repair, defense response, signal transduction, and metabolic process (Table 2). The expression patterns of *OsRAD51A1* (Os11g0615800), *Piz-t* (Os06g0286700), *Disease Resistance protein* (*DR*) (Os09g0357400), *20S PROTEASOME ALPHA SUBUNIT E1* (*OsPAE1*) (Os11g0615700), and *OsFbox341* (Os07g0158900) genes were analyzed in 2-week-old *OsNAC14*^*OX*^ plants grown in normal conditions through qRT-PCR (Figure 4). Results showed that *OsNAC14* overexpression induces the expression of *OsRAD51A1, Piz-t, DR, OsPAE1*, and *OsFbox341*. To further confirm the role of *OsNAC14* in the regulation of the five induced genes, we performed another qRT-PCR on *OsNAC14* deletion mutant produced through CRISPR/Cas9 system. The mutant lacked seven nucleotides (712–718 bp) resulting in frame shift on its C-terminal region containing the NLS sequences (Figures S3, S6). Results showed that *OsNAC14* transcript was significantly reduced in *osnac14* relative to NT plants (Figure 4). In addition, expression of all the five genes induced by *OsNAC14* overexpression were significantly reduced in *osnac14* mutants confirming that *OsNAC14* is involved in the regulation of these genes.

**Table 2:** Up-regulated genes in *OsNAC14*^*OX*^ transgenic rice in comparison with non-transgenic plants.

**Gene name**	Loc_No^*^ (IRGSP)	***OsNAC14^*OX*^*/NT^†^**	*p*-value^**^
***OsNAC14***	Os01g0675800	4.0	0.02
**STRESS RESPONSE**			
*OsERF101*	Os04g0398000	2.1	0.043
*OsCIPK33*	Os11g0134300	8.2	0.039
*OsWRKY50*	Os11g0117600	4.0	0.025
***OsLEA14/WSI18***	Os01g0705200	3.2	0.039
*HSR201*	Os12g0458100	2.3	0.005
*OsRLCK66*	Os02g0212900	2.3	0.033
**DNA REPAIR**			
***OsRAD51A1***	Os11g0615800	2.0	0.001
***OsMSH4***	Os07g0486000	10.4	0.008
**DEFENSE RESPONSE**			
***Piz-t***	Os06g0286700	4.1	0.027
***Disease resistance protein***	Os09g0357400	2.5	0.018
***RPP13-like protein 3***	Os11g0590700	3.6	0.043
*Xa39*	Os11g0588600	33.7	0.007
***OsPAE1***	Os11g0615700	16.0	0.011
*OsPDR20*	Os09g0332700	2.9	0.009
*Oscyp71Z2*	Os07g0217600	2.2	0.008
**SIGNAL TRANSDUCTION**			
***OsFbox341***	Os07g0158900	11.1	0.003
*OsiWAK1*	Os11g0691100	33.6	0.044
*OsSTA127*	Os04g0521600	3.9	0.001
*OsRLCK66*	Os02g0212900	2.3	0.033
***OsCCD8a***	Os01g0566500	2.0	0.020
**METABOLIC PROCESS**			
*OsOSC11*	Os11g0562100	30.7	0.006
*OsTPS10*	Os03g0348200	2.2	0.031
*ILL8*	Os07g0249800	2.1	0.029
*OsAGP29*	Os01g0607100	2.0	0.044

* *Sequence identification numbers for the full-length cDNA sequences of the corresponding genes*.

† *The mean of duplicate biological samples*.

** *P values were analyzed by one-way ANOVA (P < 0.01). Genes discussed in the text are in boldface. These microarray data sets can be found at http://www.ncbi.nlm.nih.gov/geo/ (Gene Expression Omnibus, accession number GSE106150)*.

### Identification of genes involved in *OsNAC14* mediated drought responses

**Figure 5:**
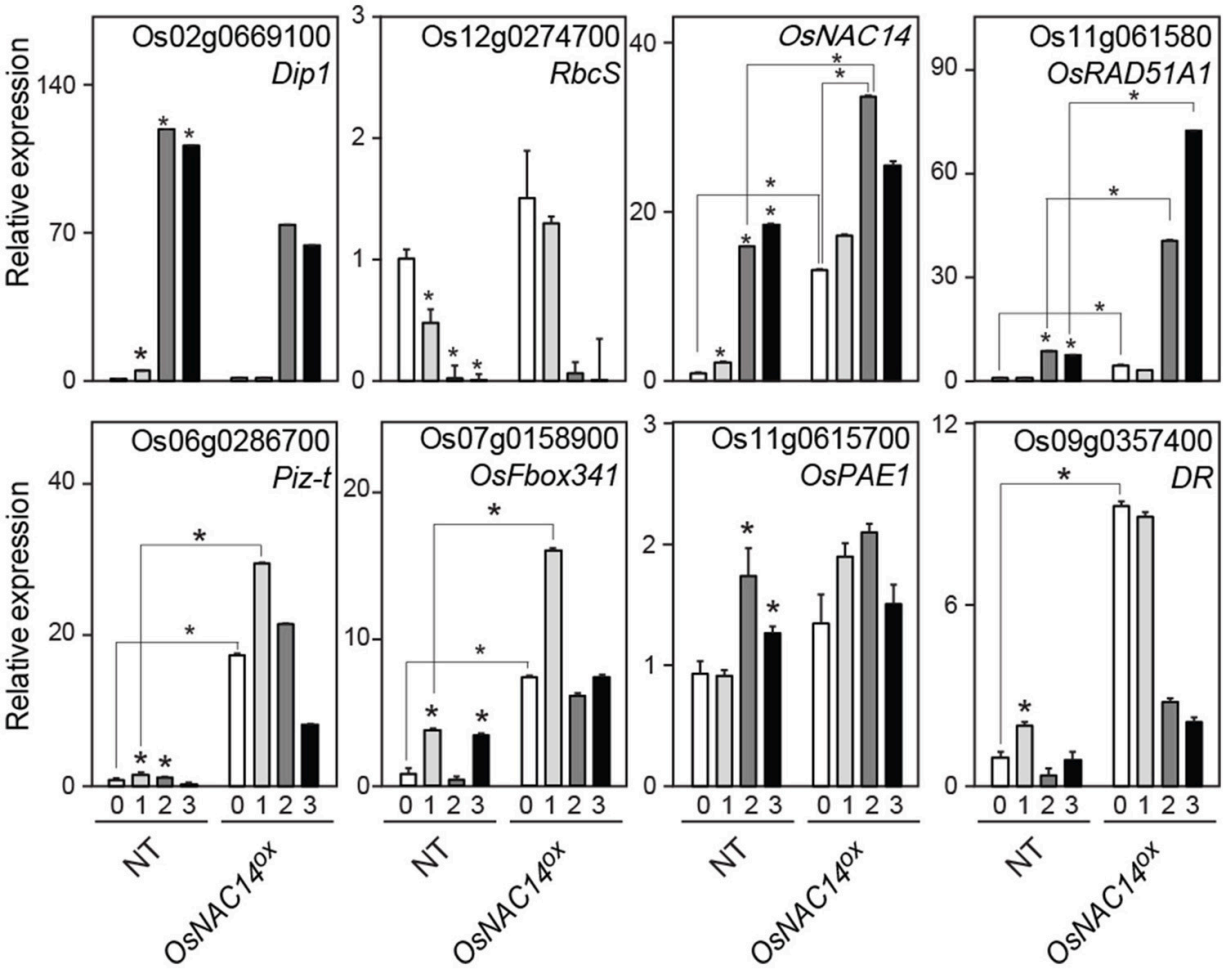
The expression pattern of *OsNAC14* dependent genes in drought conditions. Total RNAs were extracted from 4-week-old rice NT and *OsNAC14*^*OX*^ plants exposed to drought conditions for indicated period. Relative expression of tested genes was determined by qRT-PCR analysis. Expression of two drought stress marker genes, *Dehydration stress-inducible protein 1* (*Dip1*) and *Small subunit of Rubisco* (*RbcS*), was analyzed to monitor progress of drought treatment. *OsUbi1* was used as internal control for normalization. Data represent mean value + standard deviation (*SD*) (*n* = 3). Significant differences from non-treated control or between samples are indicated by asterisks (one-tailed Student's *t*-test, ^*^P < 0.05).

Next, we examined the expression pattern of five genes up-regulated by *OsNAC14* in drought conditions in order to identify downstream transcriptional network of *OsNAC14* involved in drought tolerance. qRT-PCR was performed using total RNA extracted from NT and *OsNAC14*^*OX*^ plants grown under normal and drought conditions. To confirm that drought treatment was properly applied to plants, expression of two drought-responsive marker genes, *Dehydration Stress-inducible Protein 1* (*OsDip1, Os02g0669100*) and *Small subunit of Rubisco* (*OsRbcS, Os12g0274700*), which show opposite expression patterns in response to drought stress (Jang et al., 2003) was examined. *OsDIP1* expression was induced while *OsRbcS* expression was reduced after 1 day of drought treatments validating successful induction of transcriptional reprograming turned on by drought stress (Figure 5). The induction of *OsDIP1* and reduction of *OsRbcS* in *OsNAC14*^*OX*^ plants were lower than NT plants suggesting that *OsNAC14*^*OX*^ plants are less sensitive to drought. In addition, the level of *OsNAC14* transcript induction due to drought treatment in NT was comparable with those of *PGD1* promoter under normal condition. Therefore, we aim to identify genes induced in both NT and *OsNAC14*^*OX*^ plants by drought treatment as downstream component of *OsNAC14* mediated drought tolerance pathway.

Results also showed that all five candidate genes showed higher expression levels in *OsNAC14*^*OX*^ than NT plants under both normal and drought conditions (Figure 5). However, after prolonged drought exposure (after 24 h) *Piz-t, OsFbox341*, and *DR* declined while *OsPAE1* expression was less affected by drought treatment. *OsRAD51A1*, on the other hand, was induced by both drought treatment and *OsNAC14* overexpression. Moreover, drought treatment and *OsNAC14* overexpression showed additive effects on expression of *OsRAD51A1* (Figure 5) indicating that the expression of *OsRAD51A1* is dependent on the expression of *OsNAC14*. Collectively, these results further suggest that *OsRAD51A1* is a possible direct downstream component of *OsNAC14*-mediated drought tolerance pathway.

### OsNAC14 directly regulates *OsRAD51A1*, a key component in DNA repair

**Figure 6:**
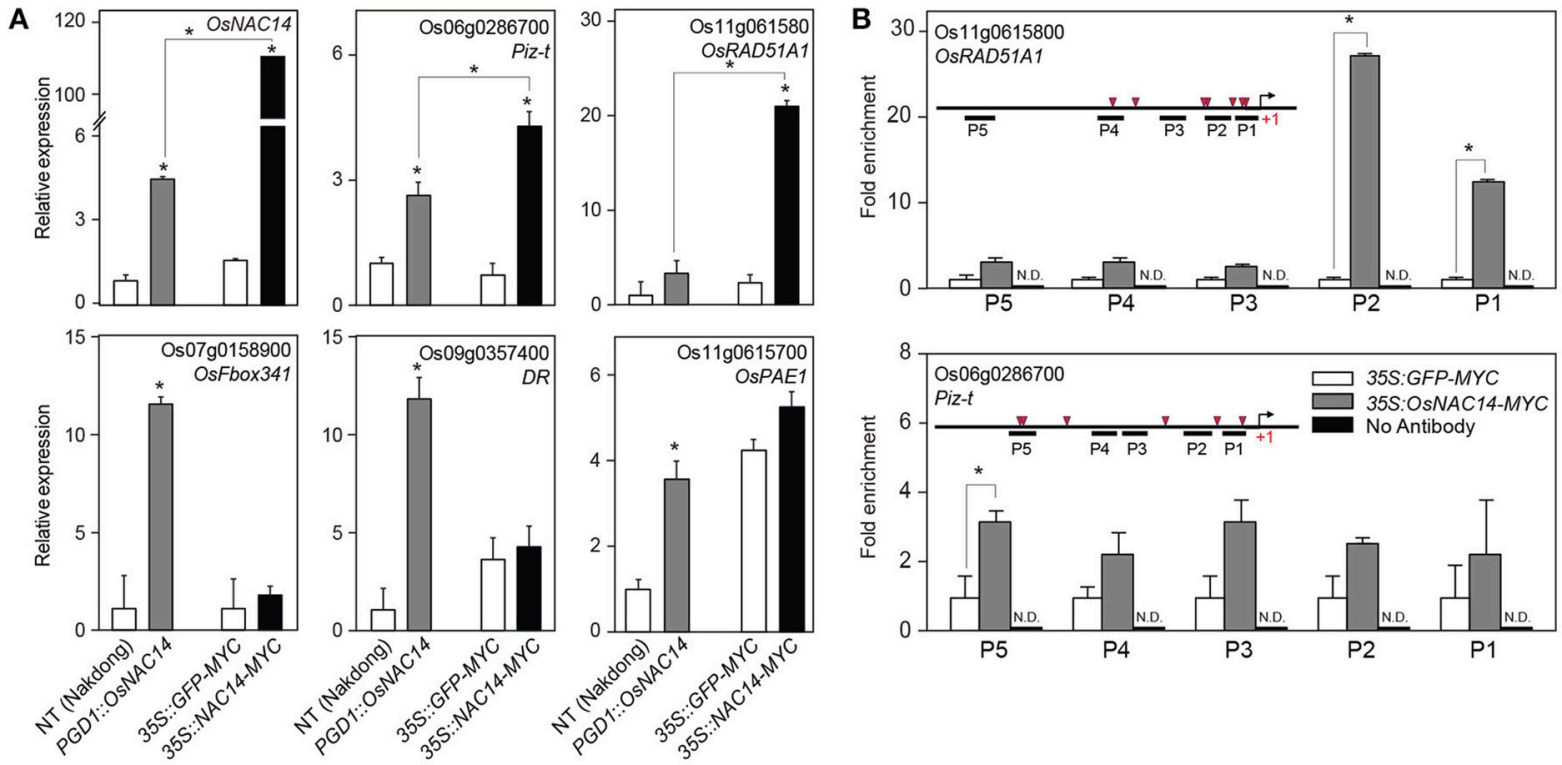
Identification of direct target gene of OsNAC14. **(A)** The expression pattern of the candidate target genes of *OsNAC14* in rice protoplasts. Rice protoplasts were isolated from 2-week-old NT and *PGD1::OsNAC14* (*OsNAC14*^*OX*^) plants (left two bars in each panel). The protoplasts isolated from NT plants were further transfected by plasmids harboring *35S::GFP* or *35S::OsNAC14* (right two bars in each panel). Total RNA was extracted from isolated protoplasts and applied for qRT-PCR analysis. *OsUbi1* was used as internal control for normalization. Data represent mean value + standard deviation (*SD*) (*n* = 3). Significant differences from NT or between samples are indicated by asterisks (one-tailed Student's *t*-test, ^*^P < 0.05). **(B)** Chromatin Immunoprecipitation (ChIP)-PCR analysis. Chromatins in rice protoplasts were precipitated using anti-MYC antibody and applied for qRT-PCR analysis. The structure of promoter and positions of tested region by qRT-PCR are illustrated in graph (left top). Red triangles represent distribution of NAC binding motif in the promoter regions. Rice protoplasts transfected with *35S::GFP-MYC* were used as negative control for *35S::OsNAC14-MYC*. ChIP experiment performed without anti-MYC antibody were applied as negative control for anti-MYC antibody. 1% input was used as control for normalization. Data represent mean value + standard deviation (**SD**) (*n* = 3). N.D., not detected. The information of primers used for ChIP-PCR was listed in Supplemental Table S2. Significant differences are indicated by asterisks (one-tailed Student's *t*-test, ^*^P < 0.05).

To confirm whether *OsRAD51A1* is a direct target of OsNAC14, we performed chromatin immunoprecipitation (ChIP) coupled with qRT-PCR analysis on rice protoplast system. Protoplasts provide a good platform to perform functional characterization of genes and isolation of protein-DNA complex in plants (Zhang Y. et al., 2011; Lee J. H. et al., 2017). We first performed qRT-PCR on total RNA isolated from protoplasts of both NT and *OsNAC14*^*OX*^ plants (Figure 6A). Similar with stable transgenic plants, all five genes were up-regulated in protoplasts of *OsNAC14*^*OX*^ plants suggesting that the isolated protoplasts maintained transcriptional network of intact plants similar to what was observed by Lee J. H. et al. (2017) (Figure 6A). Next, we introduced *35S::OsNAC14-MYC* or *35S::GFP-MYC* effector plasmids into protoplasts isolated from NT plants to verify that the transient expression of *OsNAC14* can induce similar response that was shown in stable *OsNAC14*^*OX*^ plants. As a result, *Piz-t* and *OsRAD51A1* were significantly induced in protoplasts after being transfected with *35S::OsNAC14-MYC* (Figure 6A). However, expression levels of *OsFbox341, DR*, and *OsPAE1* were not significantly increased by transient expression of *OsNAC14*. Among the five tested genes, *Piz-t* and *OsRAD51A1* were highly induced by transient expression of *PGD1::OsNAC14* compared to stable transformation. This could be due to a higher expression of *OsNAC14* in transient system but also indicates that expression of these two genes were tightly correlated with *OsNAC14* transcript level (Figure 6A). We therefore hypothesized that in addition to *OsRAD51A1, Piz-t* could also be a putative direct target of OsNAC14. To confirm this hypothesis, we performed chromatin-immunoprecipitation (ChIP) assay using transient protoplast system. Rice protoplasts transfected with *35S::OsNAC14-myc* were subjected for ChIP assay using anti-myc antibody. The *35S::GFP-myc* vector was independently transfected into rice protoplast as control. Enrichment of OsNAC14 on promoter regions was analyzed through ChIP-PCR analysis using genomic DNA isolated by ChIP assay (Figure 6B). Results showed very high enrichment on the promoter of *OsRAD51A1* but low in *Piz-t* confirming that OsNAC14 can strongly bind to the promoter of *OsRAD51A1* thereby regulating its expression (Figure 6B). Previous report has defined the 4 bps core sequence of the NAC binding motif as CACG (Olsen et al., 2005) which we found clustered in the promoter of *OsRAD51A1* while it was segregated in *Piz-t* promoter.

## Discussion

Here, we demonstrated that the rice *OsNAC14* transcription factor, a member of ONACII subgroup, is a regulator of drought tolerance pathway in rice. *OsNAC14* was induced by drought, high salinity, ABA and low temperature (Figure 1A and Figures S1B,C). Expression levels of *OsNAC14* are similar in roots and other aerial parts of plants under normal growth conditions (Figure 3); however, induced expression of *OsNAC14* by abiotic stresses was more predominant in leaves than in roots (Figure 1A and Figure S2). Other previously reported NACs belonging to the SNAC subgroup showed robust expression patterns in roots than leaves in response to abiotic stress (Redillas et al., 2012; Jeong et al., 2013; Lee D. K. et al., 2017). Thus, OsNAC14, unlike SNACs, acts as stress-induced transcription factor mainly in aerial parts of plants.

*OsNAC14* overexpression induced drought tolerance in plants both at the vegetative and the reproductive stages of growth (Figure 2, Figure S4 and Table 1). We observed that overexpression of *OsNAC14* also affected the reproductive growth of plants such as the panicle length and number of total grains under normal growth conditions (Table 1). It is possible that constitutive overexpression of *OsNAC14* may have perturbed the reproductive development through alteration of its downstream gene expression. Introducing resistance (R) gene (e.g., *RPM1* in Arabidopsis and *Pi-ta* in rice) reported to cause yield penalties in plants due to a cost of resistance (Tian et al., 2003; Wang et al., 2015). A group of R genes up-regulated by *OsNAC14* overexpression including *Piz-t* (Os06g0286700) and *RPP13-like protein 3* (Os11g059700) similarly might have affected reproductive growth of *OsNAC14*^*OX*^ plants. Nevertheless, overexpression of *OsNAC14* can confer drought tolerance at the reproductive stage as evidenced by the higher filling rate over NT controls upon stress treatments (Table 1).

The RNA-seq analysis identified 122 up-regulated downstream genes in *OsNAC14*^*OX*^ plants (Table S1). The downstream genes could be divided into four groups: stress-inducible, defense related, DNA repair, and strigolactone biosynthesis genes. The stress-inducible genes include *LATE EMBRYOGENESIS ABUNDANT PROTEIN14/Water stress induced 18* (*LEA14/Wsi18*) which has been reported to improve drought tolerance of rice by increasing of proline and soluble sugar content and cell membrane stability (Kaur et al., 2017). Thus, the up-regulation of *OsLEA14*/*Wsi18* by *OsNAC14* overexpression contributed to enhanced drought tolerance of the plants. Defense relate proteins are also closely correlated with drought tolerance in plants. It has been reported that activation of plants defense against pathogens by expressing pathogen protein elicitors or R genes induces drought tolerance (Chini et al., 2004; Peng et al., 2015; Ma et al., 2017; Wang et al., 2017). Expression of *Magnaporthe oryzae* protein elicitors, *Hypersensitive response-inducing protein 1* and *2* (*MoHrip1* and *2*), can confer drought tolerance in rice together with enhanced blast resistance (Peng et al., 2015; Wang et al., 2017). The *NUCLEOTIDE BINDING SEQUENCE- LEUCINE-RICH REPEAT* (NBS-LRR) type *ACTIVATED DISEASE RESISTANCE* (*ADR1*) and grapevine (*Vitis amurensis*) *VaRGA1* also convey significant drought tolerance and disease resistance in plants (Chini et al., 2004). It remains elusive how activation of defense responses induces drought tolerance. Overexpression of *MoHrip1* and *2* up-regulates ABA biosynthetic (*OsNCED2, OsNCED3*, and *OsZEP1*) and signaling (*OsbZIP23*) genes. Moreover, exogenous application of harpin or overexpression of an *hrf1* gene in rice promotes stomatal closure through ABA signaling (Dong et al., 2005; Zhang L. et al., 2011). Overexpression of *OsNAC14* up-regulated the expression of *NBS-LRR* type genes including *Piz-t, RPP13-like protein* and *Disease resistance protein* (Figure 4 and Table 1) that contributes to drought tolerance of *OsNAC14*^*OX*^ plants. Similarly, other *OsNAC*s involved in drought tolerance also induce expression of defense related genes. The overexpression of *OsNAC6* and *10* induces expression of *LRR* and *PATHOGEN-RELATED PROTEIN* (*PR*) genes, respectively (Nakashima et al., 2007; Jeong et al., 2010). The irreversible DNA damages generated by environmental stresses adversely affect plant growth and development. Thus, proper induction of DNA repair system in response to DNA damage facilitates plants to adapt to stress conditions (Roy, 2014). Drought stress causes DNA damage in plants (Mittler, 2002; Miller et al., 2010; Yao et al., 2013). Accumulation of ROS and oxidative damage generated by drought stress is regarded as inducer of DNA damage, such as double strand break (DSB), base deletion, and base modification (Tuteja et al., 2001; Mittler, 2002; Roldan-Arjona and Ariza, 2009; Miller et al., 2010). These lead to increased homologous recombination and mutation frequency in plants under drought conditions (Wang and Zhang, 2001; Yao and Kovalchuk, 2011; Yao et al., 2013). In this study, we found that expression of *OsRAD51A1*, a rice homolog of yeast *RAD51*, was not only highly induced under drought conditions (Figure 5) but also its expression was up-regulated by *OsNAC14*. *OsRAD51A1* expression was induced by both stable and transient overexpression of *OsNAC14* (Figures 5, 6). Moreover, expression level of *OsRAD51A1* was reduced in *osnac14* mutants (Figure 4). Our ChIP-PCR analysis revealed that OsNAC14 was bound to *OsRAD51A1* promoter containing NAC binding elements (Figure 6). Taken together, OsNAC14 binds to *OsRAD51A1* promoter and activates its expression. *RAD51* is indispensable for successful homologous recombination to repair DSBs (Shinohara et al., 1992; Li et al., 2004, 2007; Rajanikant et al., 2008) and up-regulation of *OsRAD51* increases DNA repair efficiency and alleviates cell death, thereby conferring tolerance under salinity and genotoxic stress conditions in rice (Tripathi et al., 2016). Thus, regulation of *OsRAD51A1* expression offers another molecular mechanism for drought tolerance. Collectively, *OsNAC14*^*OX*^ plants activate DNA repair system via regulation of *OsRAD51A1*, which may alleviate drought-mediated DNA damage (Figure 6). Biosynthesis of strigolactone (SL) is reported to positively regulate drought response in plants (Ha et al., 2014). Expression of *CAROTENOID CLEAVAGE DIOXYGENASE 8a* (*OsCCD8a*) was up-regulated in the *OsNAC14*^*OX*^ plants (Table 2). SL is derived from carotenoids through multiple steps of enzymatic reactions. CAROTENOID CLEAVAGE DIOXYGENASE 7/MORE AXILLARY GROWTH 3 (CCD7/MAX3) and CCD8/MAX4 are two key enzymes to cleave 9-*cis*-β-carotene to carlactone, a precursor of SL (Alder et al., 2012). Compared with WT, SL-deficient mutants (*max3* and *max4*) exhibit increased water loss rate during dehydration. This phenotype is rescued by exogenous application of SL (Ha et al., 2014), indicating that SL is required for drought tolerance in plants. Biosynthesis of SL is positively regulated by both drought and ABA in plants. The transcript of *CCD7* and *CCD8* increases by drought stress and decreases by re-watering in soybean (Song et al., 2016). In addition, ABA deficient mutants *notabilis* (mutated in NCED) and *flacca* (mutated in aldehyde oxidase) showed reduced levels of SL, together with reduced expression of *LeCCD7* and *LeCCD8* in tomato (López-Ráez et al., 2010). Moreover, exogenous application of SL enhances drought tolerance through reduced water loss and enhanced antioxidant activity (Sedaghat et al., 2017). In this study, we found that expression of *OsCCD8a* was up-regulated in the *OsNAC14*^*OX*^ plants (Table 2) that accumulates SL, leading to drought tolerant phenotype.

In conclusion, the data presented here suggest that *OsNAC14* enhances drought tolerance in rice. Specifically, *OsNAC14* directly regulates the expression of *OsRAD51A1* and regulates other downstream target genes for stress response, DNA repair, defense related, and strigolactone biosynthesis, which together confers drought tolerance in rice.

## Accession numbers

Gene from this article can be found in the National Center for Biotechnology Information (http://www.ncbi.nlm.nih.gov/) under following accession numbers: *OsNAC14* (Os01g0675800), *OsDIP1* (Os02g0669100), *OsRbcS* (Os12g0274700), *OsCIPK33* (Os11g0134300), *OsLEA14/WSI18* (Os01g0705200), *OsRAD51A1* (Os11g0615800), *OsMSH4* (Os07g0486000), *Piz-t* (Os06g0286700), *DR* (Os09g0357400), *RPP13-like protein 3* (Os11g0590700), *OsPAE1* (Os11g0615700), *OsFbox341* (Os07g0158900), *OsCCD8a* (Os01g0566500). The data set can be found at from GEO database with series accession number GSE106150 for RNA-sequencing data (http://www.ncbi.nlm.nih.gov/geo/).

## Author contributions

JSS, NO, PJC, and J-KK designed experiment and JSS, NO, and PJC performed experiments. YSK performed field experiments and analyzed yield components in field conditions. JSS, YDC, and J-KK wrote the manuscript and prepared the figures.

### Conflict of interest statement

The authors declare that the research was conducted in the absence of any commercial or financial relationships that could be construed as a potential conflict of interest.
