# Supplementary material for: Overexpression of OsNAC14 Improves Drought Tolerance in Rice
Source: Front Plant Sci. 2018 Mar 9;9:310. doi: 10.3389/fpls.2018.00310 (PMC5855183; doi:10.3389/fpls.2018.00310)
Supplement: Supplementary file 1 [file Image1.PDF]

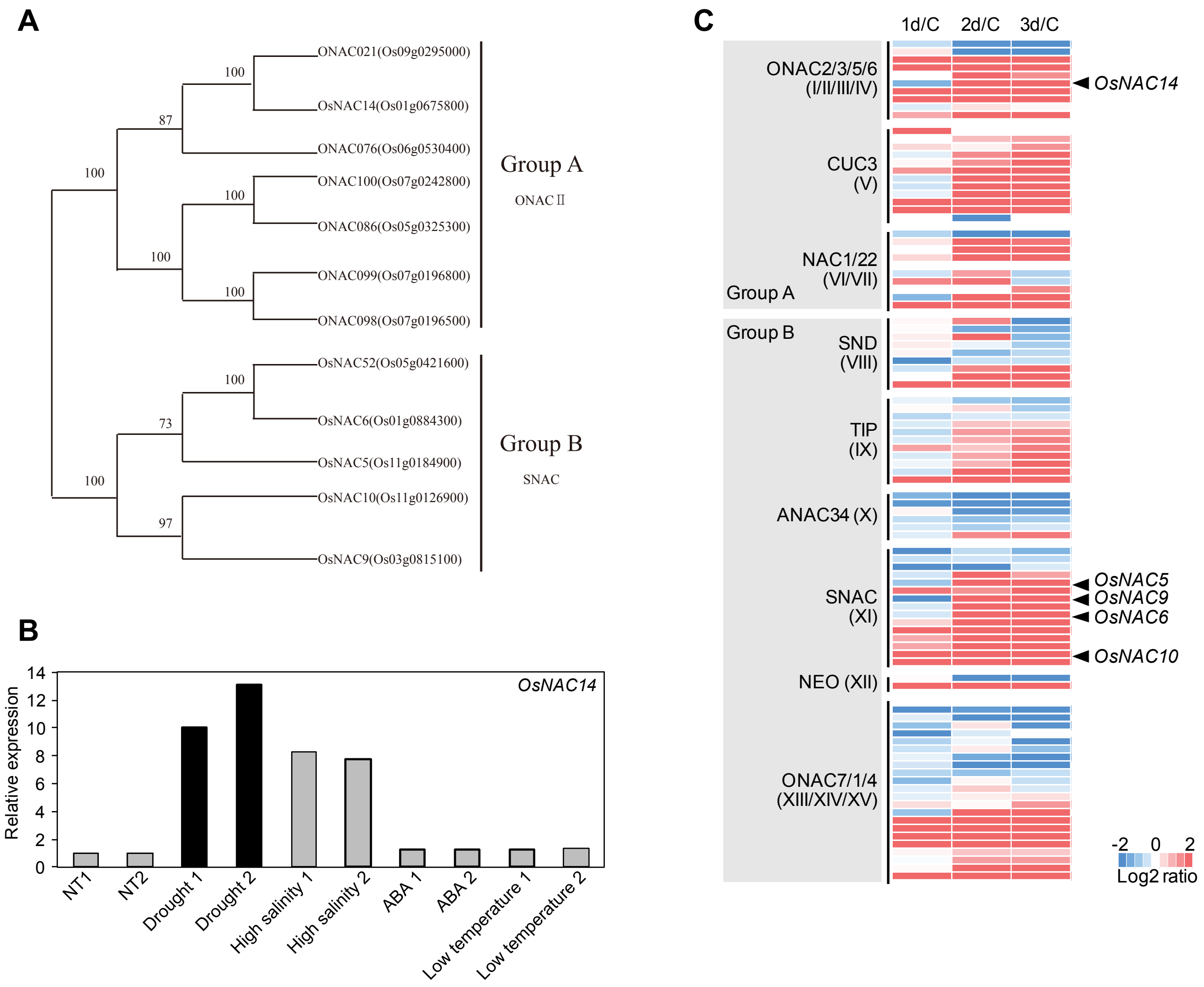

Supplementary Figure S1. Expression pattern of *OsNAC14* under abiotic stress conditions. (A) Phylogenetic analysis of OsNAC14. Multiple sequence alignment with 12 full-length OsNAC proteins was performed using ClustalW (www.genome.jp/tools/clustalw. The phylogenetic tree was constructed by the neighbor-joining method with 100 bootstrap repetitions. (B-C) The relative expression of *OsNAC14* under abiotic stresses. Expression patterns of *OsNAC14* were isolated from previously reported microarray data (B) (Oh et al., 2009) (Oh et al., 2009) and RNA-sequencing data (C) (Chung et al., 2016). Heatmap represents expression level of *OsNAC* family genes under drought stress. The bar at the bottom of the heat map represents fold change of expression induced by drought treatment.
