# Supplementary material for: Overexpression of OsNAC14 Improves Drought Tolerance in Rice
Source: Front Plant Sci. 2018 Mar 9;9:310. doi: 10.3389/fpls.2018.00310 (PMC5855183; doi:10.3389/fpls.2018.00310)
Supplement: Supplementary file 2 [file Image2.PDF]

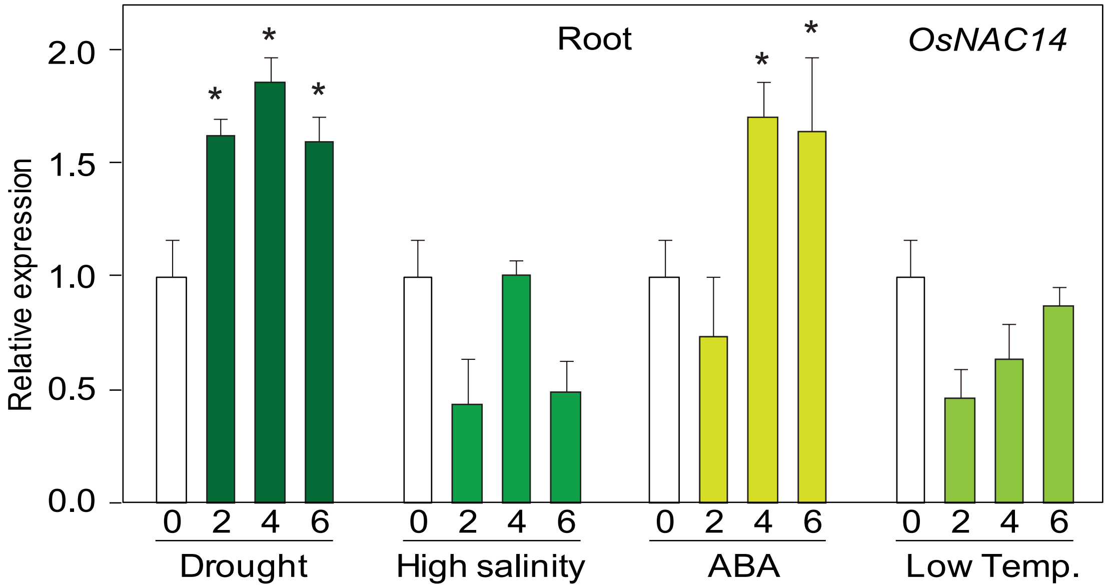


**Supplementary Figure S2. The relative expression patterns of *OsNAC14* in roots in response to four different abiotic stresses.** Two-week-old rice seedlings (Oryza sativa. L. Japonica cv. Ilmi) were exposed to air-drying (drought), 400 mM NaCl (high salinity), 100 µM abscisic acid (ABA) and 4 °C (low-temperature). Roots of rice plants were harvested at indicated time point after treatment. *OsUBIQUITIN1* (*OsUbi1*) was used as internal control for normalization. Data represent mean value + standard deviation (SD) (n=3). Significant differences from non-treated control are indicated by asterisks (one-tailed Student's *t*-test, $^{*}P<0.05$).
