# Supplementary material for: Overexpression of OsNAC14 Improves Drought Tolerance in Rice
Source: Front Plant Sci. 2018 Mar 9;9:310. doi: 10.3389/fpls.2018.00310 (PMC5855183; doi:10.3389/fpls.2018.00310)
Supplement: Supplementary file 3 [file Image3.PDF]

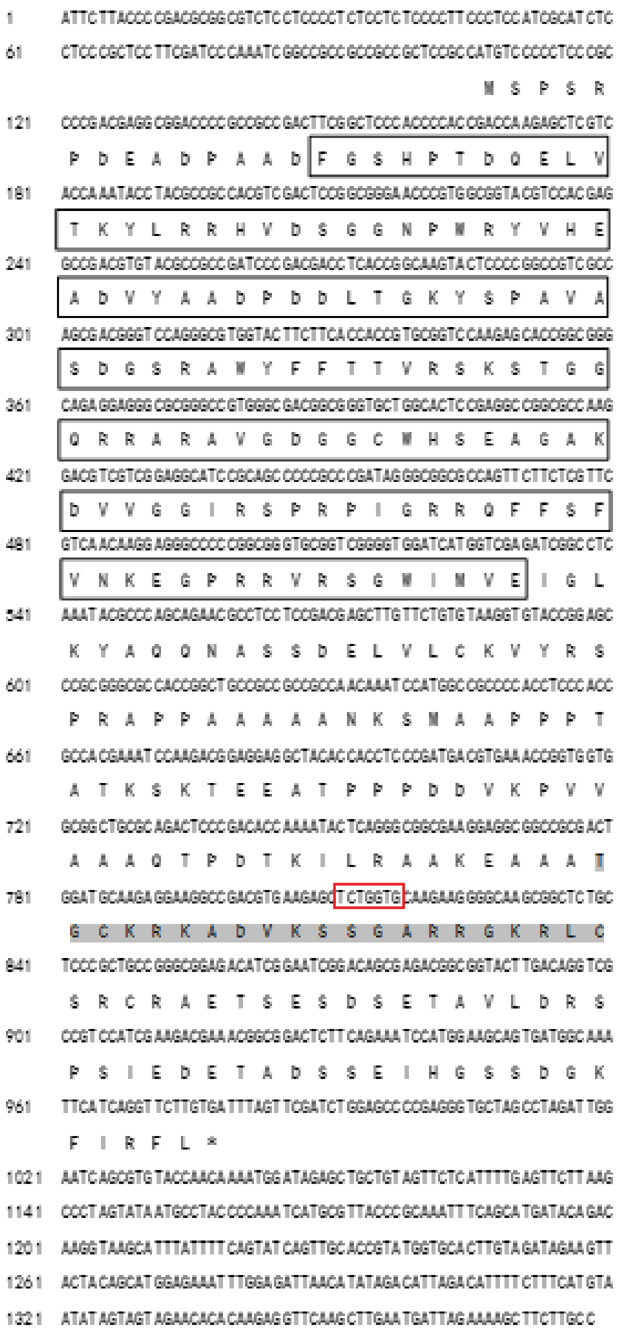

Supplementary Figure S3. Full-length coding sequence (CDS) and amino acid sequences of OsNAC14. Data represent full-length *OsNAC14* transcript. Sequence assigned with amino acid represent full -length CDS region of *OsNAC14*. Boxed sequence represents conserved NAM motif, and shaded sequence represents predicted nuclear localization signal (NLS) (Kosugi et al., 2009). The sequence boxed with red indicates position of deletion in in*osnac14* mutant plants.
