# Supplementary material for: Overexpression of OsNAC14 Improves Drought Tolerance in Rice
Source: Front Plant Sci. 2018 Mar 9;9:310. doi: 10.3389/fpls.2018.00310 (PMC5855183; doi:10.3389/fpls.2018.00310)
Supplement: Supplementary file 4 [file Image4.PDF]

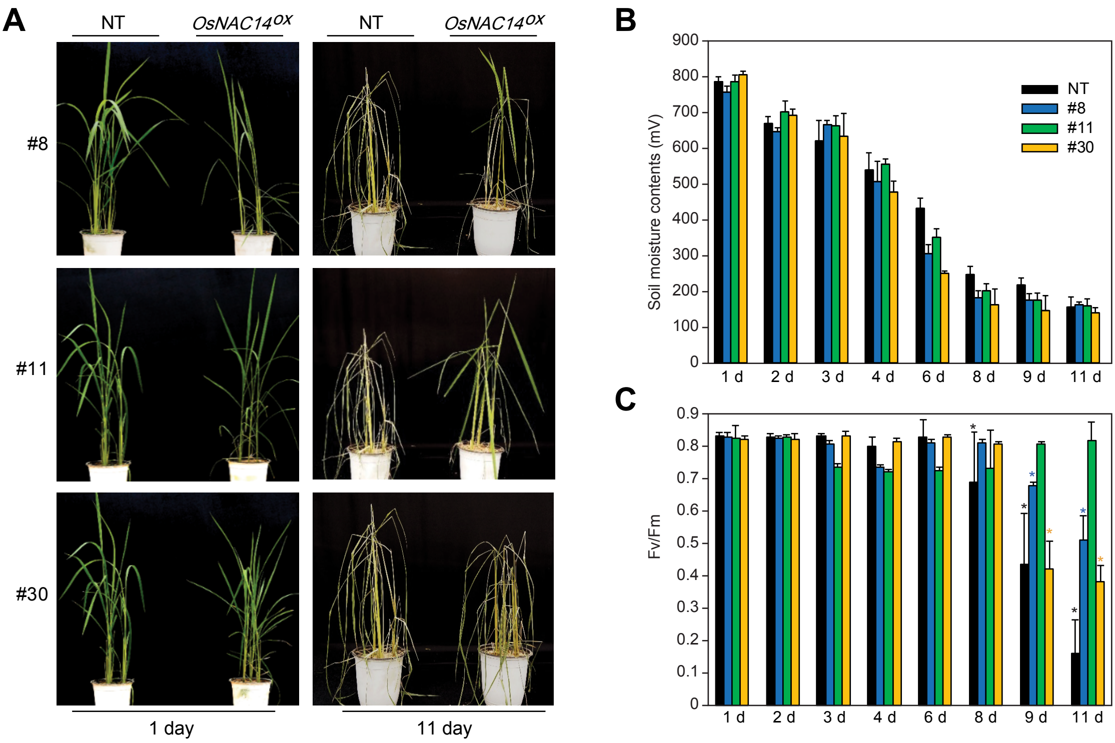

Supplementary Figure S4. Change of chlorophyll fluorescence (Fv/Fm) contents in drought conditions. (A-C) Thirty plants of 5-week-old non -transgenic (NT) and three independent homozygous *OsNAC14*^{ox} lines were exposed to drought stress by withholding water. (A) The phenotype of NT and *OsNAC14*^{ox} transgenic plants 11 days after drought treatment. (B) Measurement of soil moisture contents (mV). Each measurement was performed at different points of soil. Data represent mean +SD (n=30) (C) Chlorophyll fluorescence (Fv/Fm) contents in drought conditions. Chlorophyll fluorescence was measured in the dark at indicated time point using a Pulse Amplitude Modulation (PAM) fluorometer. Data represent mean value + SD (n=20). Significant differences from non -treated control are indicated by asterisks (one-tailed Student's *t*-test, $^{*}P<0.01$).
