# Supplementary material for: Overexpression of OsNAC14 Improves Drought Tolerance in Rice
Source: Front Plant Sci. 2018 Mar 9;9:310. doi: 10.3389/fpls.2018.00310 (PMC5855183; doi:10.3389/fpls.2018.00310)
Supplement: Supplementary file 5 [file Image5.PDF]

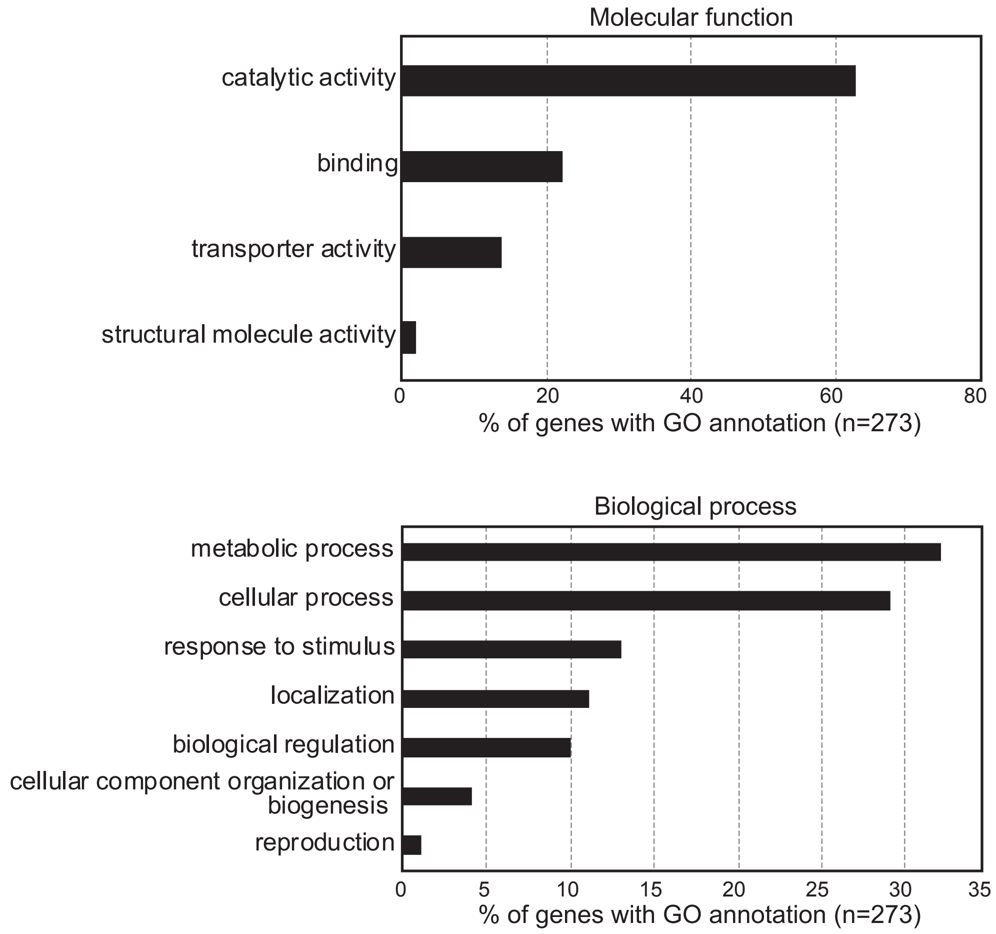

Supplementary Figure S5. Gene Ontology (GO) analysis of differentially expressed genes (DEGs) in *OsNAC14*^{ox} plants. GO analysis of the selected 273 DEGs was performed using PANTHER Classification 26 System (http://pantherdb.org). DEGs were classified into categories corresponding to molecular function and biological process.
