# Supplementary material for: Overexpression of OsNAC14 Improves Drought Tolerance in Rice
Source: Front Plant Sci. 2018 Mar 9;9:310. doi: 10.3389/fpls.2018.00310 (PMC5855183; doi:10.3389/fpls.2018.00310)
Supplement: Supplementary file 6 [file Image6.PDF]

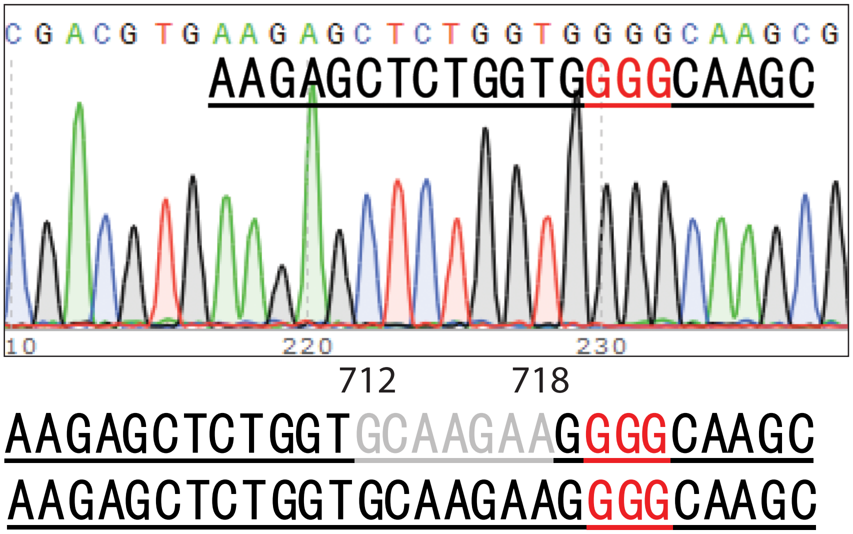

Supplementary Figure S6. Characterization of *osnac14* deletion mutant plants generated through *CRISPR/Cas9* system. 2-week-old non-transgenic (NT) and *osnac14* mutant plants were harvested and used for genomic DNAextraction. 7 nucleotides (712-718bp) in OsNAC14 were deleted in *osnac14* mutants. Grey: position of deletion, Red: position of PAM site.
